# Photo-Micrography

**Published:** 1900-03

**Authors:** 


**Photo-Micrography.** By Edmund J. Spitta. Pp. xi., 163. London: The Scientific Press, Limited. 1899.

A thoroughly scientific and at the same time an eminently practical treatise is given to us in this handsome quarto. We especially like the arrangement of the several chapters; devoting a separate chapter to low, medium, and high-power work is a very good method of arrangement. The chapters on the microscope and lens, and on the substage condenser, are remarkably lucid and clear, and will be found as useful by microscopists as by photographers.

After reading the chapter on Illuminants, one comes to the conclusion that the use of limelight is a necessity for photo-micrographic work with the high powers of the microscope: but humbler workers in the art who have not the power of providing themselves with the necessary apparatus for this need not despair of getting excellent results with other means; *e.g.*, we have seen excellent photographs of bacteria taken with the incandescent gaslight, and also some taken with magnesium wire. This latter means of illumination is not mentioned in the book.

The book does credit to publishers as well as author, and we cordially recommend it to all workers on the subject. The reproductions of photo-micrographs in the plates at the end of the work are beautiful.

